# Recent Jishishan earthquake ripple hazard provides a new explanation for the destruction of the prehistoric Lajia Settlement 4000a B.P.

**DOI:** 10.1038/s41598-024-60433-8

**Published:** 2024-05-21

**Authors:** Peijun Shi, Fenggui Liu, Xingmin Meng, Qiang Zhou, Deyong Yu, Qiong Chen, Lianyou Liu, Weihua Fang, Cunde Xiao, Chunyang He, Tao Ye, Jinpeng Hu, Ying Li

**Affiliations:** 1 State Key Laboratory of Earth Surface Processes and Resource Ecology, Beijing Normal University, Beijing, 100875 China; 2 Academy of Plateau Science and Sustainability, Qinghai Provincial People’s Government-Beijing Normal University (Qinghai Normal University), Xining, 810016 China; 3 College of Geological Sciences and Mineral Resources, Lanzhou University, Lanzhou, 730000 China; 4 Academy of Disaster Reduction and Emergency Management, Ministry of Emergency Management-Ministry of Education (Beijing Normal University), Beijing, 100875 China

**Keywords:** Environmental social sciences, Natural hazards

## Abstract

The Jishishan Ms 6.2 earthquake occurred at 23:59 on December 18, 2023 in Gansu Province, China. We conducted a field survey to assess the hazards and damages caused by the earthquake and its associated geo-activities. Subsequently, we organized a seminar to discuss the possible causes of the destruction of a prehistoric site—Lajia Settlement—dated back to four thousand years B.P. and located only several kilometers away from the epicenter of the Jishishan earthquake. The Jishishan earthquake was unique for its hazard and disaster process, which featured ground shaking and a series of complex geological and geomorphological activities: sediment and soil spray piles, liquefaction, collapse, landslide, and mudflow along water channels. We define this phenomenon as the Jishishan earthquake ripple hazard (JERH). The most recent evidence from the JERH suggests that a prehistoric earthquake similar to the JERH, instead of riverine floods or earthquake-induced landslide dam outburst flood, as previously hypothesized, destroyed the Lajia Settlement.

## The Jishishan Earthquake

**Figure 1:**
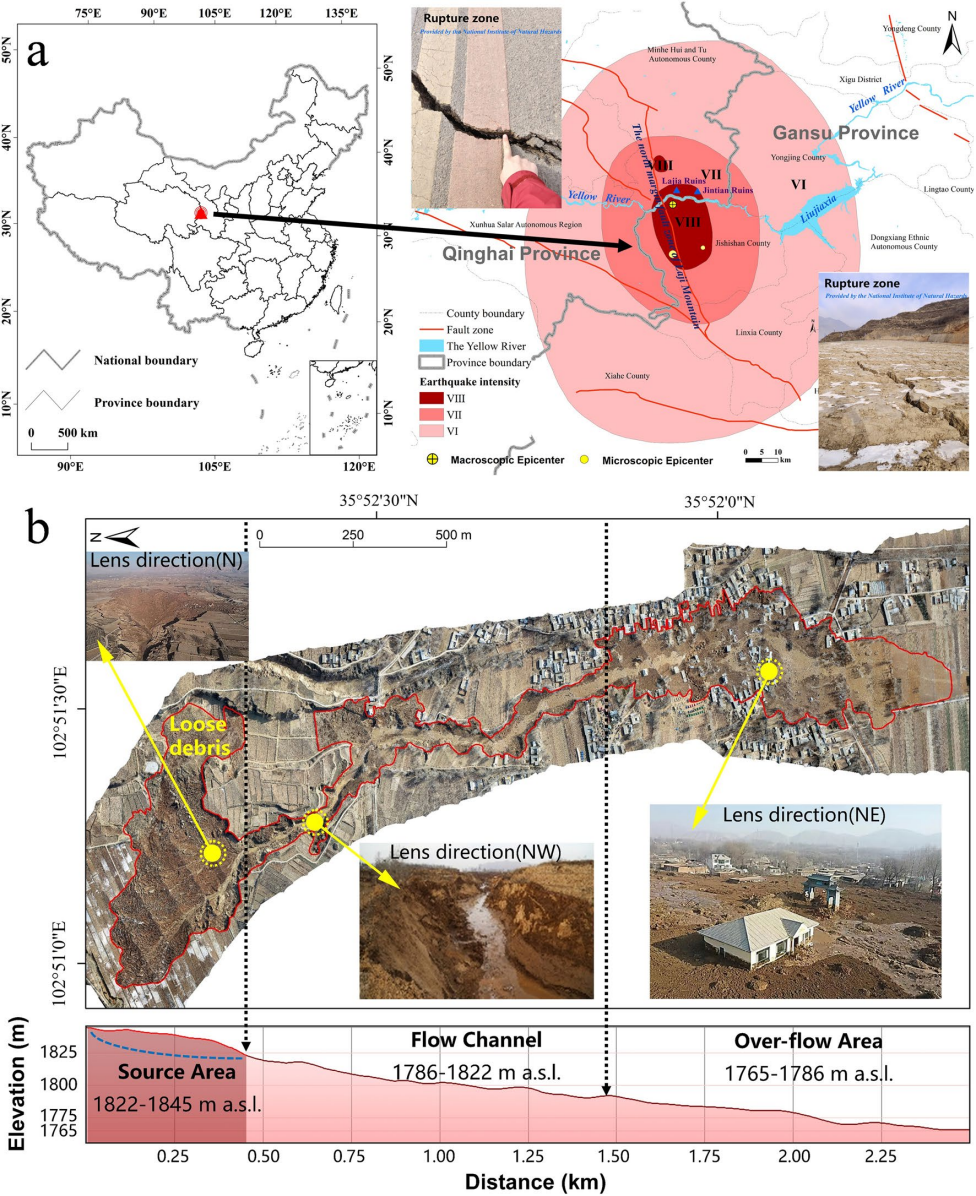
The earthquake ripple hazard caused by the Ms 6.2 earthquake in Jishishan County, Gansu Province in China. (**a**) Location and seismic intensity distribution of the earthquake in Jishishan County in China; (**b**) Earthquake ripple hazard area in Jintian and Caotan Villages in Zhongchuan Township, Minhe County, Qinghai Province in China, based on remote sensing imagery and on-site photographs. (Note: The map was generated using ArcMap 10.6 software, URL: https://www.arcgis.com/index.html, by co-authors).

On December 18, 2023 at 23:59, a Ms 6.2 (Mw5.9) earthquake (epicenter 35.70° N and 102.79° E) struck Jishishan County, Gansu Province, China. The long axis of the isoseismic line was 124 km in the North-northwest direction, and the short axis was 85 km. The maximum intensity was VIII with a total area of 331 km^2^, and the area with the intensity of VI was 8364 km^2^ (Fig. 1a)^1^. The Holocene fault zone on the northern margin of the Lazhi Mountains is in the north-northwest direction and passes through Jintian and Caotan Villages in Zhongchuan Township of Minhe Hui and Tu Autonomous County, Qinghai Province (Fig. 1a). Here, the earthquake caused a horizontal displacement of 4 cm, a vertical displacement of 8 cm, and a crack width of 10–15 cm (Supplement 1). Although the intensity of the earthquake in Jishishan was not exceptionally high, it caused serious casualties and property losses in the Jintian and Caotan Villages, which are located on the terraces of the main stream of the Yellow River. The western side of Jintian and Caotan Villages is only 4.15 km away from the of prehistoric Lajia Settlement in Guanting Town (Figs. 1b, 3), Minhe County (Fig. 1b), which was also damaged to some degree in this earthquake, resulting in the loss of some important cultural relics. This event attracted widespread attention from various sectors of the society.

## Jishishan earthquake ripple hazard (JERH) and disasters

Earthquake ripple hazard refers to the simultaneous occurrence of underground confined water upwelling, soil liquefaction, collapse, landslide, and mudflow triggered by ground shaking from an earthquake, jointly causing casualties, property losses, and ecological damage. Previous studies have attributed this type of complex seismic hazard chain to the liquefaction of the water-saturated soil layer induced by the earthquake^2,3^. A large number of landslides in previous earthquakes was considered related to seismic-induced liquefaction, which include the 1920 Mw 8.5 Haiyuan earthquake in Ningxia, China^4^, the 2018 Mw 6.6 Palu-East Ibri earthquake in Indonesia, the 2018 Mw7.5 Palu earthquake in Indonesia, the 2018 Mw 7.0 Lombok earthquake in West Nusa Tenggara Province, Indonesia, and the 2022 Mw 6.1 Pasaman earthquake in the Pasaman Mountains of West Sumatra, Indonesia^5–7^. Earthquake ripple hazards are more complex earthquake hazard chains that occur under specific geological and geographic environment conditions. They are different from the serial hazards formed by earthquake-induced liquefaction of water-saturated soils, and are more destructive, often resulting in “low intensity hazard and big disaster”, which requires great attention for regional disaster risk reduction, particularly in global geo-environmental transition (transformation) zones (Supplement 2).

### Initiation of the Jishishan earthquake ripple hazard

**Figure 2:**
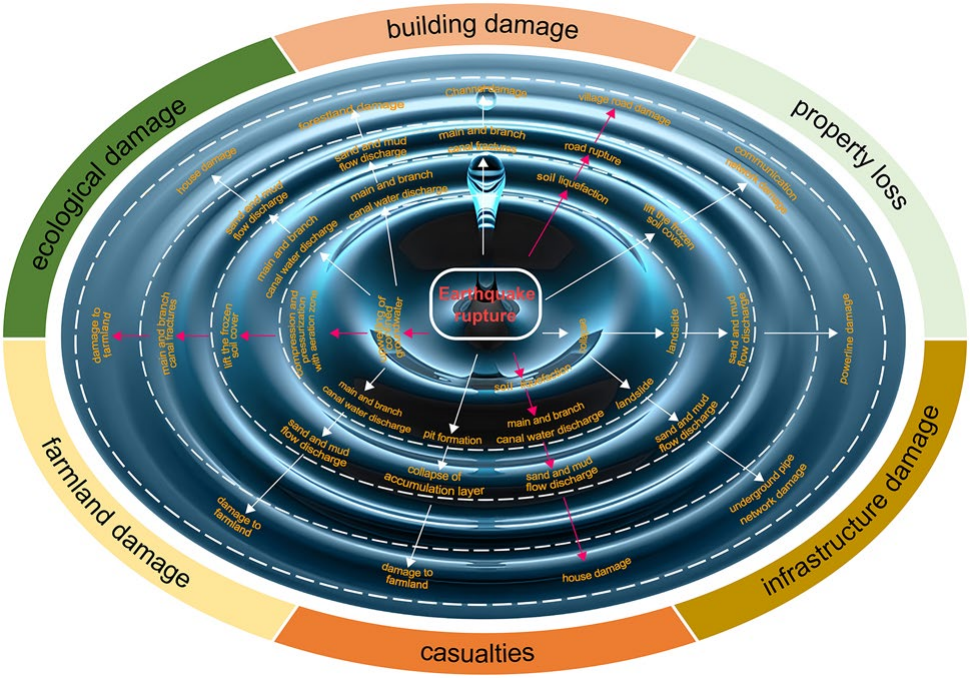
Schematic diagram of the processes of the Jishishan earthquake ripple hazard. The red arrowed lines represent three concurrent seismic hazard chains that were likely to have destroyed the Lajia Settlement.

The epicenter of the Jishishan earthquake is in the transitional zone between the Qinghai-Tibet Plateau and the Loess Plateau. The earthquake occurred in winter, when the farmland on the second and third terraces (1765–1845 m a.s.l.) of the Yellow River had been irrigated through artificial canals and a layer of frozen soil or ice of 10–20 cm had formed. The depth of the saturated loess layer associated with earthquake-induced soil liquefaction is mainly about 14 m below the terrace surface (Supplement 3). The earthquake triggered the upwelling of confined water, which compressed the gas in the upper vadose zone and broke through the surface frozen layer, resulting in a powerful sediment and soil spray. This spurt accumulated a 1.5–5.0 m thick mixed sand and soil of approximately 107,500 m^3^ in volume, covering an area of 33,000 m^2^. The large pit left by the spurt caused the surrounding soil mass to collapse and slide, forming a 6–10 m high landslide. The landslide, composed of surface soil, red soil, and lower layer of sandy loess, moved downward rapidly under the combined effect of earthquake-induced liquefied mudflow and gravity, initiating the JERH (Fig. 2) (Supplement 4). We estimated that the volume of eroded material in the source area is about 604,900 m^3^ (Supplement 5).

### Rapid change and discharge of source material

The sand and mud flow slid rapidly and eroded and widened the existing valley (Qijia Valley). It also incorporated more soil material from the lateral collapse of the ravine walls. The terraced slopes facilitated the movement of the unstable soil mass and the release of high-concentration sand and mud flows. The water and ice in the irrigation canals and farmland were also involved in the slide and discharge. The flow velocity of the debris reached up to 2.0–3.5 m/s, causing a rapid replenishment for the debris flow with a linear length of 2.8 km. This flow not only destroyed farmland and trees, but also damaged roads, houses, powerlines, communication networks (towers), pipe networks, and so on (Supplement 5).

### Debris flow diffusion and reduced flow

Although the sliding and sediment flows were greatly attenuated by friction, they still formed a 3–5 m high head of splashes when they reached the downstream residential, farmland, and forest areas on the second terrace. They covered an area of 181,800 m^2^; the height of deposition ranged from 0.5 to 5.0 m, resulting in an accumulated volume of 675,000 m^3^ (Supplement 5).

### Earthquake ripple hazard and disaster loss and damages

The JERH caused 20 deaths, and destroyed 93 houses, some roads, powerlines, communication networks, pipe networks, irrigation canal networks, and other infrastructure. It also damaged a large tract of forestland, farmland, and ecosystems.

## A novel explanation of the cause of destruction of the ancient Lajia Settlement

**Figure 3:**
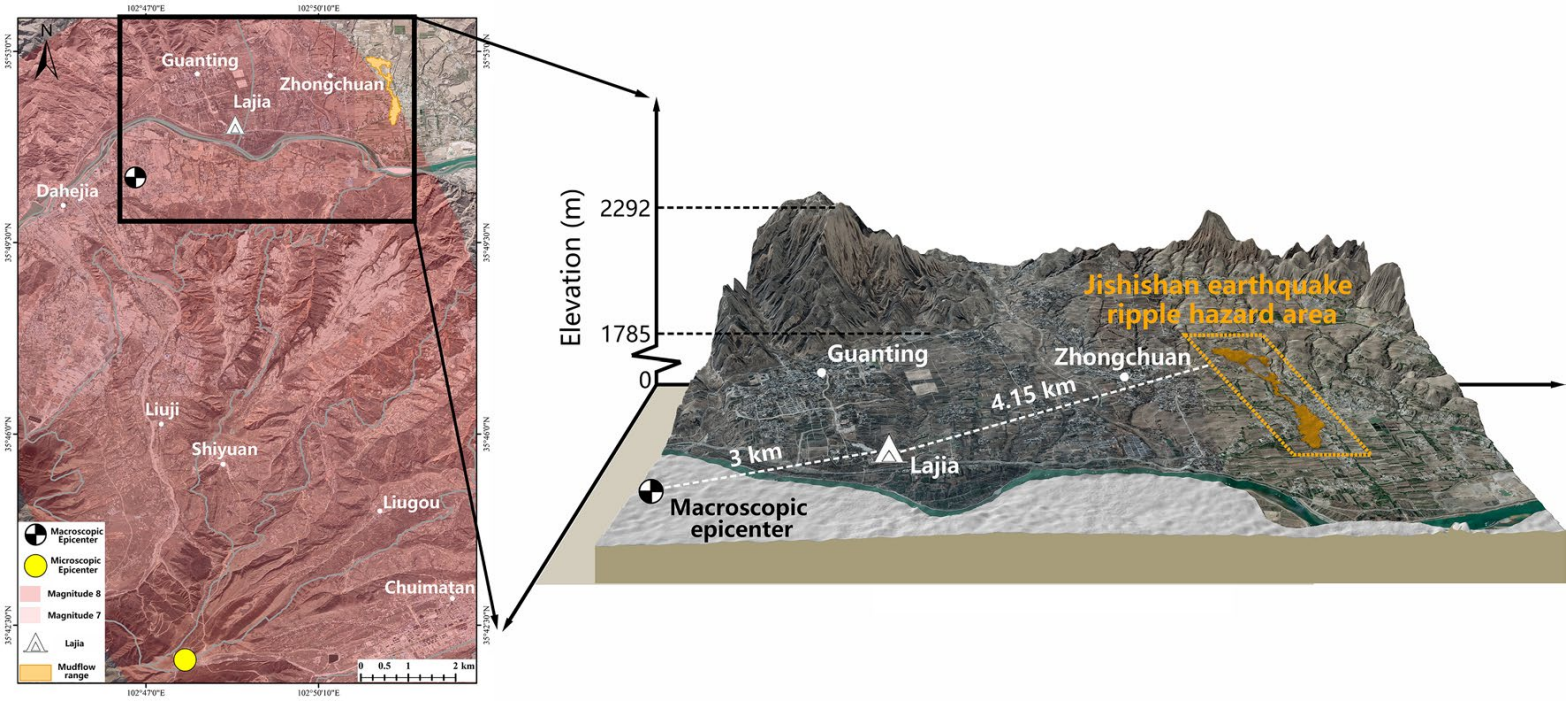
Location of the Jishishan earthquake ripple hazard (JERH) area and the Lajia Settlement from 4000a B.P., Minhe County, Qinghai Province in China. (Note: The map was generated using ArcMap 10.6 software, URL: https://www.arcgis.com/index.html, by co-authors).

The distance between Jintian and Caotan Villages of the JERH area and the Lajia Settlement (dated 4000a B.P.) is 4.15 km. They are both located in the Guanting Basin in the upper reaches of the Yellow River, on the second and third terraces of the Yellow River (Fig. 3). The two places have the same geological and geographic environment.

### Controversies over the cause of destruction of the Lajia Settlement

The discovery of the prehistoric disaster site of Lajia in the Guanting Basin of Qinghai Province in the upper reaches of the Yellow River, especially the tragic scene of a mother protecting her child with her body, is shocking^8^. The cause of the destruction of the Lajia Settlement is widely agreed to be an earthquake. However, the origin of the red clay layer that enveloped the human remains and killed the population has become the focus of debate, sparking intense academic controversies^9–17^. Some scholars believe that this clay layer is the evidence of a catastrophic Yellow River flood^9,10^; others believe that the clay layer came from a dammed lake flood after an earthquake that created a barrier lake in Jishixia^11^. A third view is that this clay layer was a mudflow deposit, and that the direct cause of the disaster was a sudden torrential rain and mudflow after the earthquake^13,17^.

After Wu et al.^10^, Dong et al. argued that the prehistoric disaster at the Lajia site had nothing to do with the Yellow River flood^12^. Wu et al.^15^ believe that Wu et al.’s^10^ arguments are unconvincing because the physical evidence linking the prehistoric disaster at the Lajia site to the Yellow River flood is unreliable. Han believes that the conclusions of Wu et al.^10^ on the scale of the barrier lake, the age of the lake, and the peak flow at the dam break point and Lajia site are unverifiable. And the so-called Great Flood and its impact on the formation of early Chinese dynasties have not been confirmed^16^. Huang et al. believe that Wu et al.’s^10^ hypothesis of a huge outburst flood related to the Dayu myth is an alternative explanation that ignores many published studies^14^. However, these inferences and arguments are based on local evidence from environmental archaeology, and there is still no consensus on the hazard process of the disaster. The Jishishan earthquake provides an excellent opportunity for us to reconstruct the process of the disaster at Lajia based on the present JERH.

### Interpretation of the red clay deposit associated with the destruction of the Lajia Settlement

The Lower Lajia Village of Guanting Town, where the prehistoric Lajia Settlement was located, is adjacent to Jintian and Caotan Villages in Zhongchuan Township, and situated in the northern part of the Guanting Basin on the left bank of the main stream and second terrace of the Yellow River. To the north of these two places is the third terrace of the Yellow River and middle and low mountains and hills, and the surface sediments in these two areas are composed of farmland soil, upper red soil, and lower sandy loess. Both places had developed gullies originating from the northern middle and low mountains and hills or the third terrace of the Yellow River. The gullies flow into the Yellow River from north to south and are all seasonal. The mixed sediments associated with the destruction of the Lajia Settlement was completely consistent with that of the Jishishan earthquake ripple hazard deposition of sediment and mudflow in Jintian and Caotan Villages, which is a mixture of farmland soil, sandy loess, and red soil (Supplements 4 and 6).

### Archaeological excavation scene and scale of the Lajia Settlement

The total area of the three excavated sections of the Lajia Settlement was about 0.47 km^2^, with 35 houses (including 1 pottery kiln). The site had found the remains of 25 human bodies that died in a sudden hazard event had been found at the site. Although the extent of the damage caused by the JERH in Jintian and Caotan Villages was larger than that at the Lajia site, the loss difference is believed to be related to the scale of the settlements and population, and the magnitude of the two disasters can be similar. Compared with today’s Jintian and Caotan Villages, the Lajia Settlement, which existed more than 4000 years ago, was quite “developed” at that time, but still smaller.

### Explanation of the death scene at the Lajia Site Excavation

The scene of a mother protecting her child with her body, discovered presented at the excavation site of the Lajia Settlement, was very similar to the scene of three adults protecting two children found at the JERH search and rescue site. This demonstrates the suddenness of the disaster and similar situations of harm to human lives, and indicates that the posture of instinctively holding and protecting the children when a mudflow poured into the room was more common than in the case of an earthquake and house collapse.

With the approach of “inferring the past from the present” and based on the review and analysis of the JERH and the Lajia Settlement disaster, we argue that the destruction process of the Lajia Settlement 4000a B.P. was similar to the JERH today in Jintian and Caotan Villages. It was a cluster of events involving earthquake-induced ruptures, confined water upwelling and soil liquefaction, collapse, landslide, and mudflow. It was likely that an earthquake near the Lajia Settlement at that time, with a magnitude and intensity comparable to today’s Jishishan earthquake, caused similar geological and geomorphological activities and ultimately resulted in the destruction of the Lajia settlement. Due to the limitations in field observation and the large scale of the ripple hazard associated with the Jishishan earthquake, it is difficult to show the entire area involved in the disaster in a single picture, therefore we use three on-site photos as supplementary material (Supplement 7).

## Implications for disaster prevention, mitigation, and relief in the geographic transitional zone

The earthquake ripple hazard in Jishishan was a typical process of “low intensity hazard and big disaster” in the transitional zone between the Qinghai-Tibet Plateau and the Loess Plateau in western China. It was a compound of concurrent occurrence of earthquake, rupture, confined water upwelling and soil liquefaction, collapse, landslide, and mudflow. The affected area is earthquake-prone and has rich loess deposits, on a terrace of the Yellow River where groundwater level is high, and is routinely irrigated for farmland moisture conservation in winter. The spatial overlap of these environmental factors was the culprit causing this hazard and disaster process.

For this reason, the damage process is on a par with the earthquake-induced confined water upwelling and liquefaction, collapse, landslide, and mudflow, and the significant difference between them as a concurrent disaster chain and a cascading disaster chain should be clarified. We believe that the seismic ripple damage caused by the Ms 6.2 earthquake in Jishishan County, Gansu Province, occurred in the transition zone between the Qinghai-Tibet Plateau and the Loess Plateau, which has certain implications for us to deal with the complex, chain generation and concurrent disasters occurring in similar geographical environment transition zones. For example, the Qinghai-Tibet Plateau and the Yunnan-Guizhou Plateau, the Qinghai-Tibet Plateau and the Inner Mongolia Plateau, the Qinghai-Tibet Plateau and the Sichuan Basin have also experienced similar Jishishan earthquakes in history (the Ms 7.7 Tonghai earthquake in Yunnan Province in 1970, the Ms 8.0 Gulang Earthquake in Gansu Province in 1927, and the Ms 8.0 Wenchuan earthquake in Sichuan Province in 2008). In the transitional zone between the sea and land across the world, it is necessary to pay special attention to the ripple hazards and disasters initially caused by a single event, composed of multiple factors and processes of the Earth, multi-scale compounds and superpositions, that is, multi-hazards clusters.

The ripple hazards and disasters in the geographic transitional zones of western China are also often coupled with high exposure and high vulnerability of human activities^18^, as well as the increasing frequency of climate and weather extremes driven by global climate change. These changes bring new and severe challenges to the security of the human society. Therefore, a deep understanding of the “low intensity hazard and big disaster” caused by the earthquake ripple hazard in the transitional zone between the Qinghai-Tibet Plateau and the Loess Plateau not only provides a scientific explanation for the cause of the destruction of the prehistoric Lajia Settlement, but also offers new insights for human beings to deal with the ripple hazards in such environments.

### Supplementary Information

Supplementary Information.

## Data Availability

All data during this study are included in this article and its supplementary information files.
