# Supplementary material for: Recent Jishishan earthquake ripple hazard provides a new explanation for the destruction of the prehistoric Lajia Settlement 4000a B.P
Source: Sci Rep. 2024 May 21;14:11630. doi: 10.1038/s41598-024-60433-8 (PMC11109126; doi:10.1038/s41598-024-60433-8)
Supplement: Supplementary file 1 — Supplementary Information. [file 41598_2024_60433_MOESM1_ESM.docx]

**Supplement Materials for**

**“Recent Jishishan Earthquake Ripple Hazard Provides a New Explanation for the Destruction of the Prehistoric Lajia Settlement 4000a B.P.”**

Peijun Shi^1,2,4 *^, Fenggui Liu^2^, Xingmin Meng^3^, Qiang Zhou^2^, Deyong Yu^1^, Qiong Chen^2^, Lianyou Liu^1,4^, Weihua Fang^1,4^, Cunde Xiao^1^, Chunyang He^1,4^, Tao Ye^1,4^, Jinpeng Hu^1,4^, Ying Li^1,4^

1 State Key Laboratory of Earth Surface Processes and Resource Ecology, Beijing Normal University, Beijing 100875, China.

2 Academy of Plateau Science and Sustainability, Qinghai Provincial People's Government–Beijing Normal University (Qinghai Normal University), Xining 810016, China.

3 College of Geological Sciences and Mineral Resources, Lanzhou University, Lanzhou 730000, China.

4 Academy of Disaster Reduction and Emergency Management, Ministry of Emergency Management–Ministry of Education (Beijing Normal University), Beijing 100875, China.

*Correspondence author. Email: spj@bnu.edu.cn

After the Ms 6.2（Mw 5.9） earthquake in Jishishan County, relevant government agencies organized various expert teams. Our research teams, organized by the Expert Committee of the National Disaster Reduction Commission of China, were led by the Qinghai Provincial People’s Government–Beijing Normal University Academy of Plateau Science and Sustainability (Qinghai Normal University, Beijing Normal University) and Lanzhou University. Liu Fenggui's team, Shi Peijun's team, and Meng Xingmin's team conducted field surveys and obtained data on the seismic faults, groundwater level, material composition, and amounts of soil spray and deposition in different sections of the earthquake ripple hazard area in Jintian and Caotan Villages (JTCT) of Zhongchuan Township, Minhe County, Qinghai Province, China, caused by the Ms 6.2 earthquake in Jishishan County.

**Supplement 1:** **The Ms 6.2 earthquake in Jishishan County triggered ruptures in JTCT of Zhongchuan Township, Minhe County, Qinghai Province**

In the source area of materials of the seismic ripple hazard caused by the Ms 6.2 earthquake in Jishishan County, Gansu Province, China, the horizontal surface displacement measured on site was about 4 cm, the vertical displacement was about 8 cm, and the crack width reached 10–15 cm (Fig. S1).

a b c d


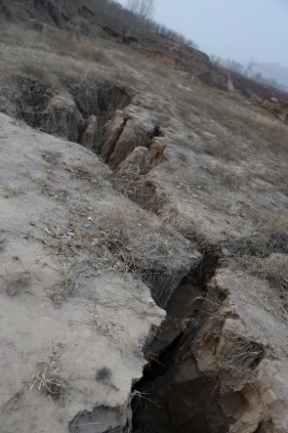

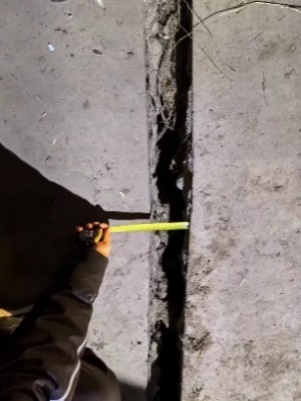

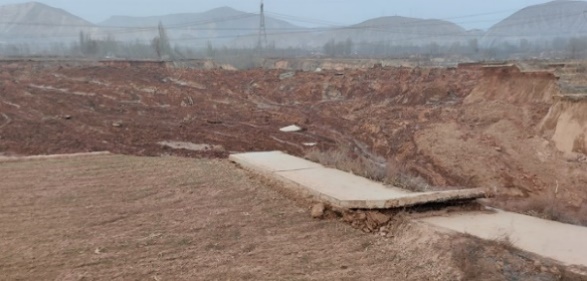

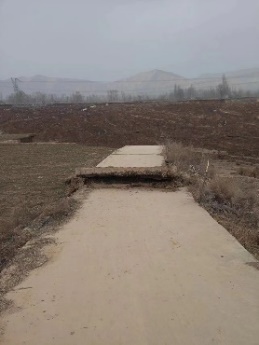


**Fig. S1 The Jintian and Caotan Village (JTCT) earthquake fault in Minhe County, Qinghai Province, China, triggered by the Ms 6.2 earthquake in Jishishan County, Gansu Province (photograph by Liu Fenggui and Shi Peijun’s teams, December 27, 2023)**

(a) A 10–15 cm wide crack in farmland; (b) A 8–10 cm wide crack on a road; (c) Vertical displacement of the road of about 8 cm; (d) Horizontal displacement of the road of about 4 cm.

This result is consistent with the InSAR co-seismic deformation field simulation results released by the Institute of Geophysics, China Earthquake Administration on December 19, 2023 at 4:50 (Fig. S2).


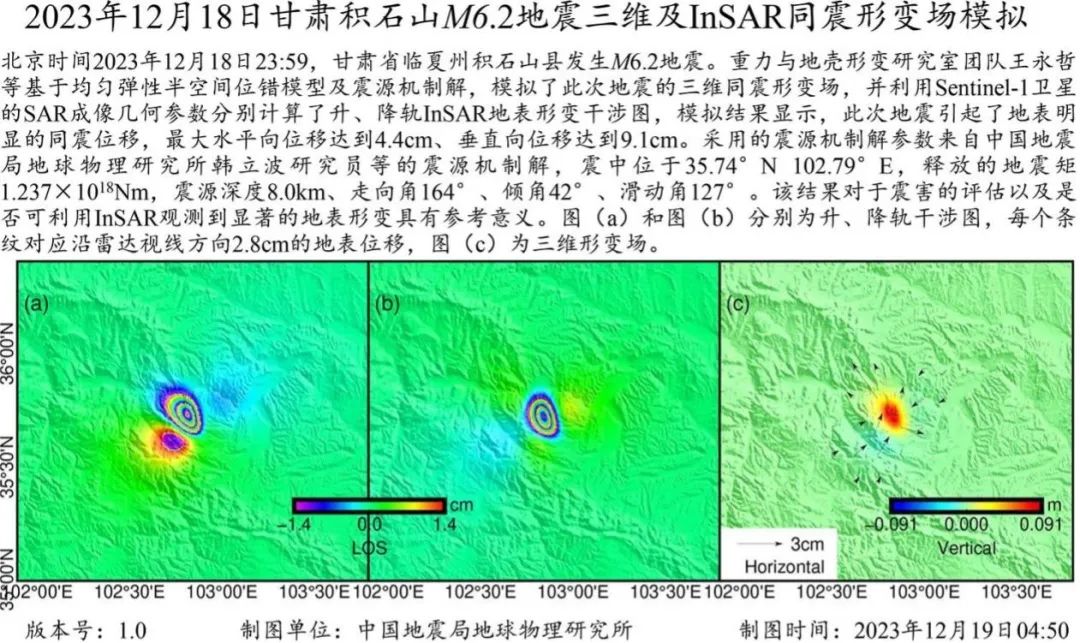


Figures (a) and (b) are the interferograms of the ascending and descending orbits, respectively, with each grave corresponding to a surface displacement of 2.8 cm along the direction of the line of sight, and Fig. (c) is a three-dimensional deformation field.

**Fig. S2 InSAR co-seismic deformation field simulation results (Institute of Geophysics, China Earthquake Administration, December 19, 2023)**

On December 18, 2023 at 23:59 Beijing time, an Ms 6.2 earthquake occurred in Jishishan County, Linxia Prefecture, Gansu Province. The research team of Wang Yongzhe from the Gravity and Crustal Deformation Research Laboratory simulated the three-dimensional co-seismic deformation field of this earthquake based on the uniform elastic half space dislocation shape and source mechanism solution. They also used Sentinel-1 satellite SAR imaging geometric parameters to calculate the InSAR surface deformation interferograms for the ascending and descending tracks. The simulation results show that this earthquake caused significant co-seismic deformation on the surface, with the maximum horizontal displacement reaching 4.4 cm and the vertical displacement reaching 9.1 cm. The parameters of the seismic source mechanism solution used are from the seismic source mechanism solution of Han Libo, a researcher at the Institute of Geophysics, China Earthquake Administration. The epicenter is located at 35.70°N and 102.79°E, and the released seismic moment is 237×10^18^ Nm. The depth of the seismic source is 8.0 km, with a strike angle of 164°, an inclination angle of 42°, and a sliding angle of 127°. This result has reference significance for the assessment of earthquake damage and whether significant surface deformation can be observed using InSAR.

**Supplement 2: Ripple hazard**

Ripples describe the propagating small waves on the water surface blown by the wind, also commonly used as a metaphor for subtle mental activities. Ripple hazard refers to a phenomenon where multiple hazards occur in groups, caused by a single hazard in a specific geographic environment (hazard-prone environment), which is referred to as a concurrent hazard chain in the literature*(19)* (Fig. S3)


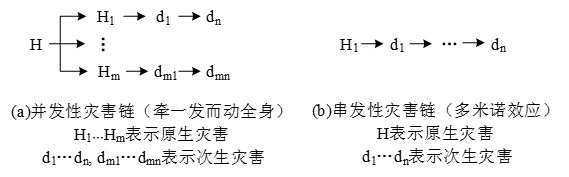


(a) (b)

1. Concurrent hazard chain (one trigger affects the whole)

H_1_,...,and H_m_ represent the primary hazard; d_1_–d_n_ and d_m1_–d_mn_ denote the secondary hazards.

1. Cascading hazard chain (domino effect)

H_1_ represents the primary hazard; d_1_– d_n_ stand for the secondary hazards.

**Fig. S3 Diagram of ripple hazard (concurrent hazard chain) and cascading hazard chain**

**Supplement 3: Groundwater level measurement**

The distribution of groundwater level was obtained by laying high-density electrical survey lines along the third terrace of the landslide (Fig. S4). It was found that the stable and continuous groundwater level is located 14 m below the surface, which is consistent with the depth at which the landslide was destabilized (Fig. S5a). However, there are discontinuous local high water content soil layers distributed within about 5 m of the ground surface in different parts of the rear edge of the landslide (Fig. S5b), which should be related to the winter irrigation carried out before the earthquake in the area (Figs. S5c, S5d). The long-term irrigation activities in the area may be the main cause of the elevated shallow groundwater level. According to the investigation, from 1969 to present, the water lifting irrigation project along the upper reaches of the Yellow River has caused a large amount of water to infiltrate into the originally dry loess layer of this area, forming a continuous and continuously rising groundwater table, providing favorable conditions for the formation of the co-seismic liquefaction-landslide-mudflow disaster chain. In addition, the area was in the period of surface freezing before the earthquake, with a frozen depth of about 10–20 cm. The outlets of groundwater (including spring water) at the foot of the slope and in the channels should have been frozen and blocked, causing the groundwater level inside the slope to rise, increasing the thickness and possibility of liquefaction of the slope.


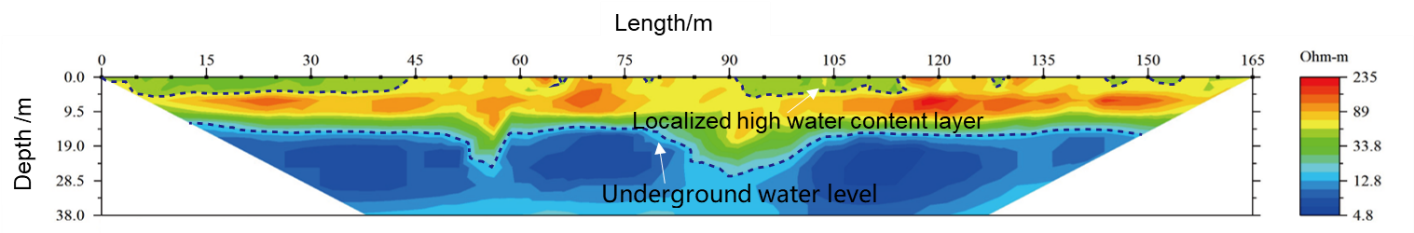


**Fig. S4 Groundwater distribution of the tableland near the landslide (December 27, 2023, by Meng Xingmin’s team)**


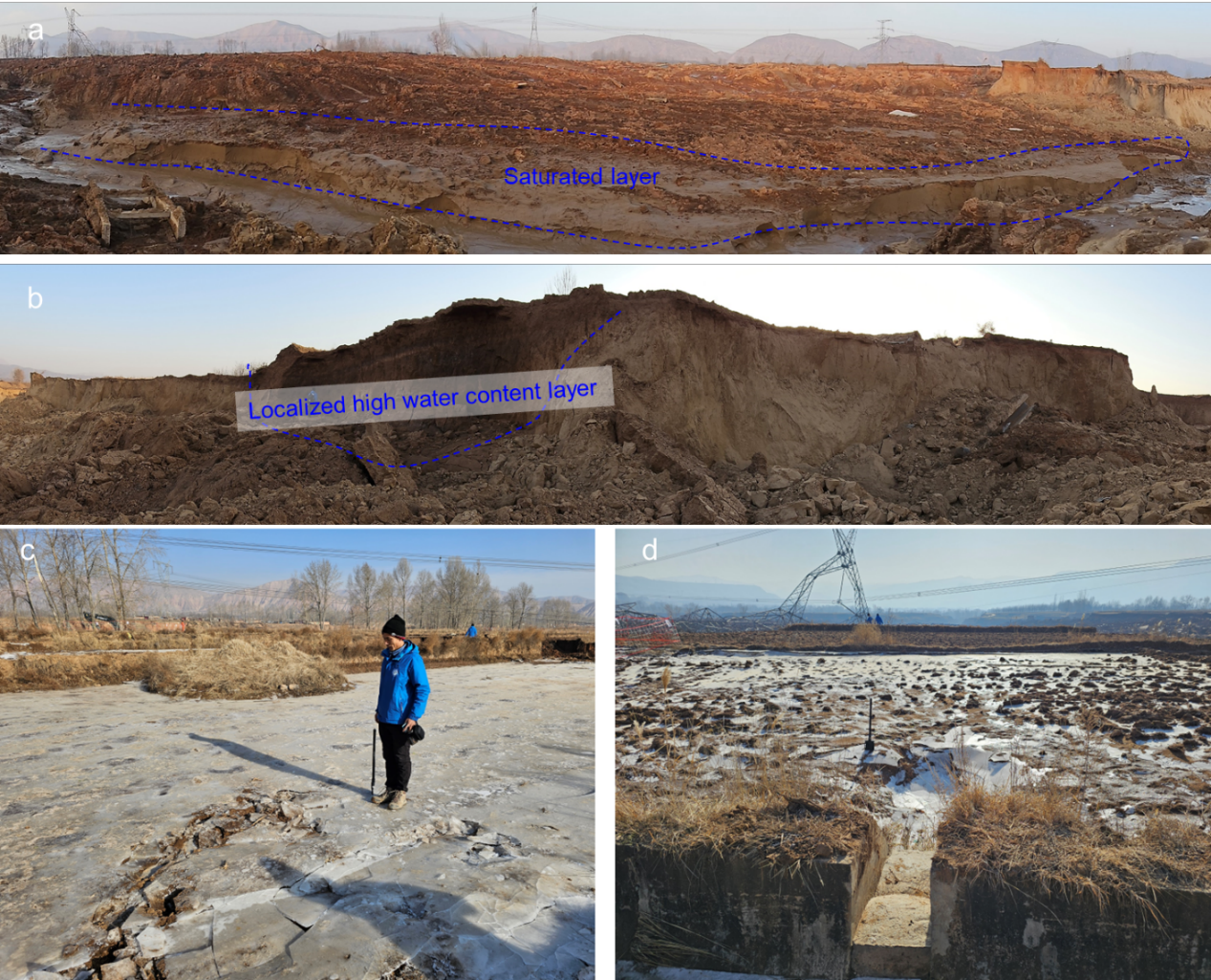


**Fig. S5 (a) Continuous saturated layer; (b) Local saturated zone; (c), (d) Iced layer caused by irrigation (photograph by Meng Xingmin’s team, December 27, 2023).**

Note: Figure. S5 Photos are provided by the co-authors with informed consent to publish these photos, and the people appearing in the images are used as a scale for comparative observation.

**Supplement 4: Material composition**

From the perspective of material composition, most of the frozen flowing mud in the source and accumulation areas is loess (Figs. S6a, S6b), with fewer red clay nodules, indicating that the main material for liquefaction and mudflow is saturated loess. There is a large amount of unsaturated red clay material accumulated at the front edge of the landslide in the source area and the bank of the flow area (Figs. S6c, S6d), indicating that the red clay overlying the loess is mostly in an unsaturated state. The unsaturated red soil layer is scattered at the rear edge of the landslide (Figs. S6e, S6f), indicating that the material movement in the front of the landslide caused the gradual collapse of the material in the rear plateau. Some of the collapsed soil material slid along with the lower saturated loess layer, and some of the material was incorporated into the fluid for flow. Under the action of earthquakes, liquefaction of saturated loess is an important mechanism that triggers such ripple hazard and disasters.


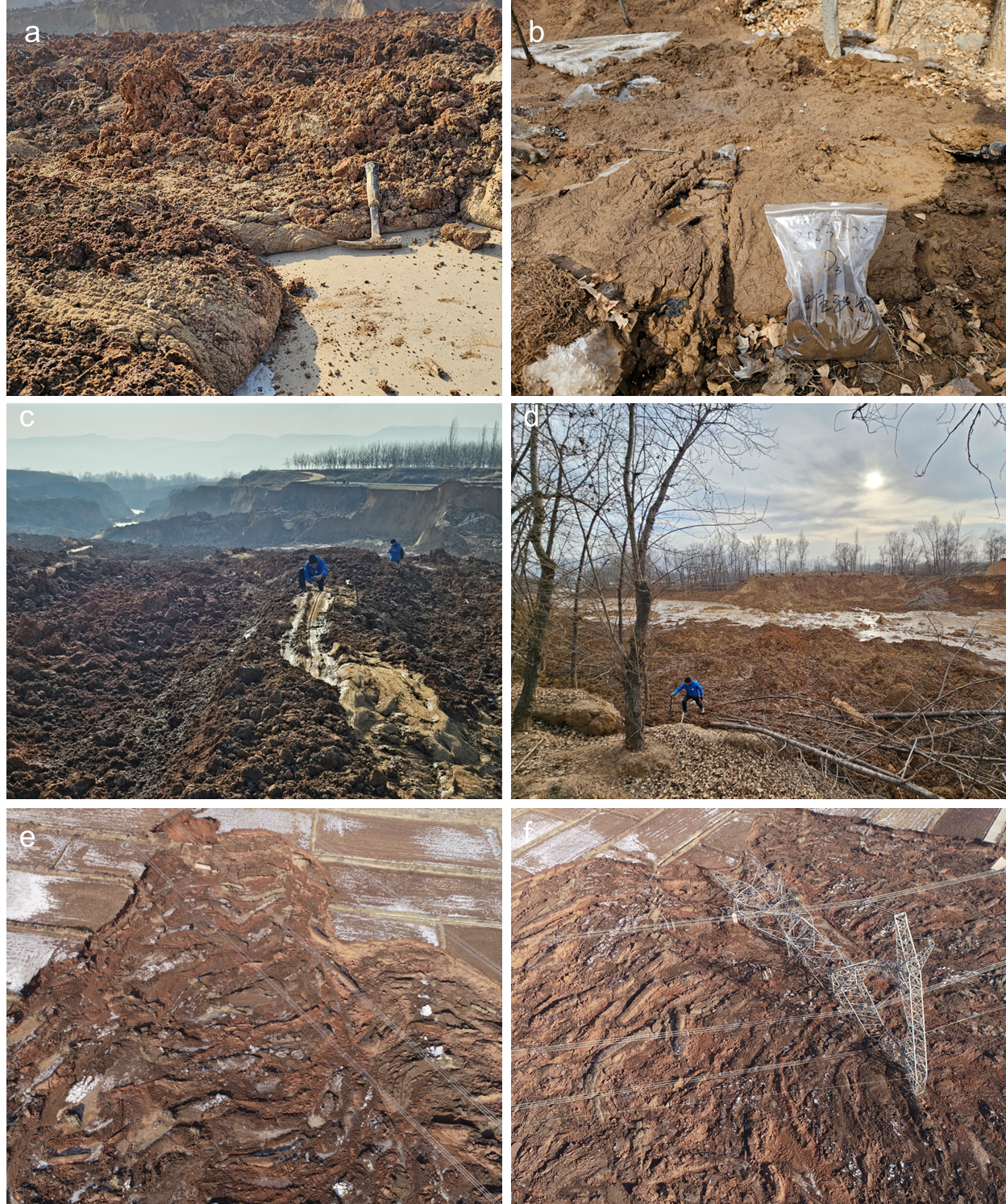


**Fig. S6 Different parts of the landslide-mudflow area. (a) Loess flow in the source zone; (b) Loess flow in the deposition zone; (c) Unsaturated red soil on the surface of the source zone; (d) Unsaturated red soil on the bank of the transport zone; (e, f) Graded terraces made of red soil (photograph by Meng Xingmin’s team, December 27, 2023).**

**Supplement 5: Measurements of displaced soil mass volume at different sections of the earthquake disaster site in Jintian-Caotan Villages, Zhongchuan Township, Minhe County, Qinghai Province, caused by the Ms 6.2 earthquake in Jishishan County**

The earthquake ripple hazard caused by the Ms 6.2 earthquake in JTCT, Zhongchuan Township, occurred at an elevation of 1765–1845 m, with the lowest air temperature generally ranging from -10 ℃ to -14 ℃ and an average daily temperature of -3.3 ℃. The winter irrigated farmland soil and seasonal stream channels on the second and third terraces of the Yellow River formed a layer of frozen soil or ice of 10–20 cm. The earthquake triggered the upwelling of confined water, which compressed the gas in the upper vadose zone and broke through the surface frozen layer, resulting in a powerful soil spray. This spurt accumulated a 1.5–5.0 m thick mixed sand and soil of approximately 107,500 m^3^ in volume, covering an area of 33,000 m^2^ (indicated by the red arrow in Fig. 7). The large pit left by the spurt caused the surrounding soil mass to collapse, forming a 6–10 m high landslide. The landslide, composed of surface soil, red soil, and lower layer of sandy loess, moved downward rapidly under the combined effect of earthquake-induced liquefied mudflow and gravity, initiating the earthquake ripple hazard (Fig. S7).


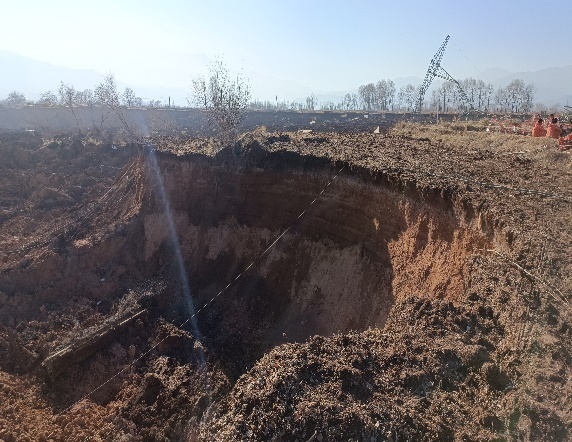


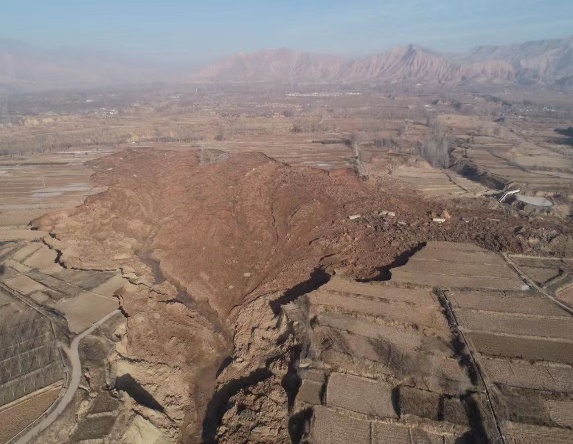


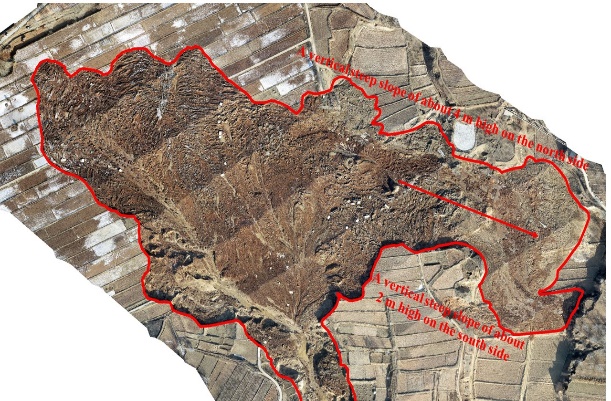

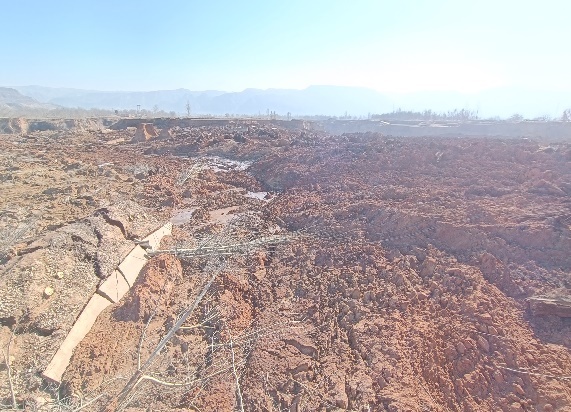


**Fig. S7 Source area of the earthquake ripple hazard in JTCT, Minhe County, Qinghai Province, caused by the Ms 6.2 Jishishan earthquake (photograph by Liu Fenggui's and Meng Xingmin’s team, December 27, 2023).**

**Note: The red arrow points to the soil spurt accumulation area.**

In the transport zone of the earthquake ripple hazard area, due to the freezing of stream water, the friction of the channel was reduced, resulting in a higher speed of flow of the ice-rich debris. The sand and mud flow slid rapidly and eroded and widened the existing valley. It also incorporated more soil material from the lateral collapse of the ravine walls. The terraced slopes facilitated the movement of the unstable soil mass and the release of high-concentration sand and mud flows (Fig. S8). The water and ice in the irrigation canals and farmland were also involved in the slide and discharge. According to the local disaster victims, it took about 10 minutes from the time of earthquake occurrence for the mudflow to enter the houses (the affected residential buildings on the second terrace slope), across a distance of 2.8 km. It is inferred that the flow velocity of the debris reached up to 2.0–3.5 m/s.


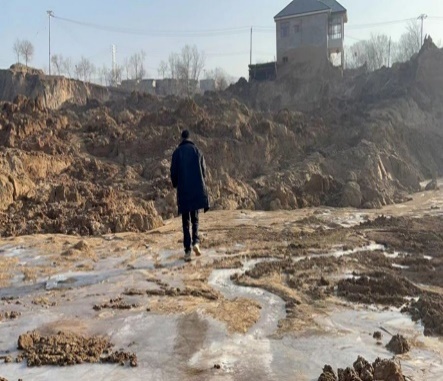

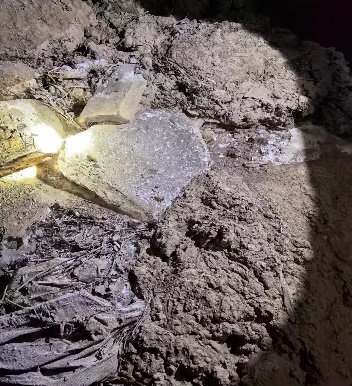

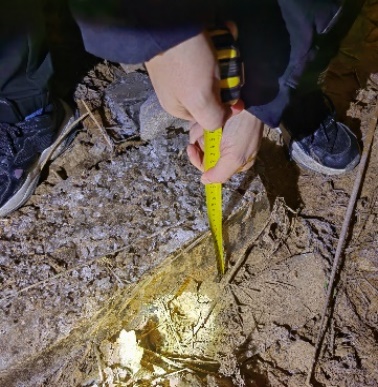


**Fig. S8 Sand and mud flow with ice blocks in the transport zone of the earthquake ripple hazard area in JTCT, Zhongchuan Township, Minhe County, Qinghai Province, affected by the Ms 6.2 Jishishan earthquake (photograph by Shi Peijun’s team and Liu Fenggui’s team, December 23, 2023)**

In the deposition section of the earthquake ripple hazard area, the sliding and sediment flow are significantly weakened by friction and gravity, and the sediment and mud flows are rapidly diffused and reduced in the terraced residential, farmland, and forest areas of the second terrace (Fig. 9).


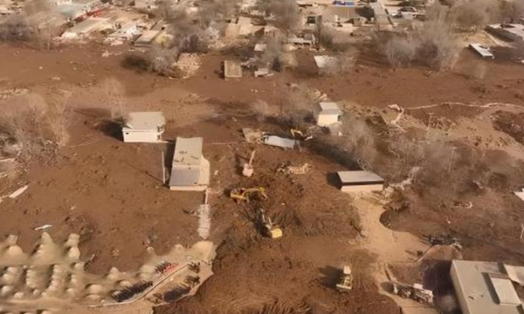
**
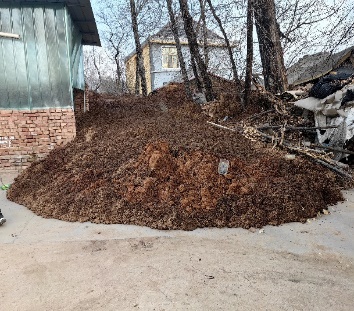
**
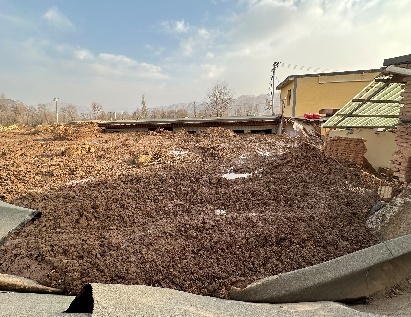


**Fig. S9 Sand and mud flow in the deposition section of the earthquake ripple hazard area in Zhongchuan Township, Minhe County, Qinghai Province (photograph by Liu Fenggui's team, December 23, 2023)**

According to the residents near the landslide source area, when they felt the earthquake and ran out of their houses, they heard a thunderous sound. The frequency of this thunder was high at the beginning, but quickly slowed down. Therefore, it is inferred that saturated loess liquefaction and landslides occurred immediately after the arrival of the seismic shear waves. About 10 minutes after the earthquake (according to the residents in Jintian Village), the mudflow reached the downstream CTJT. Based on this estimation, the average flow velocity of the mudflow was approximately 3.0 m/s. After rushing out of the middle reaches and merging into the main channel, the mudflow was blocked by the bank at the turning point of the channel at the confluence, causing it to climb and form local deposition(Fig. S10a). A large number of tall trees planted in the gully were pushed down/uprooted, indicating that the local flow velocity of the mudflow was very high. After the mudflow reaching the downstream villages, a 3–5 m high head splash was formed, burying a large number of houses with a burial area of 181,800 m^2^, a thickness between 0.5 and 5.0 m, and a cumulative burial volume of 675,000 m^3^(Fig. S10c). The frozen layer of the channel in the transport zone and the nearly 10 -15cm surface ice layer reduced the friction of the channel(Figs. S11a, Fig. S11b), resulting in higher flow velocity of the mudflow in this section. According to the digital elevation model of the sliding bed before and after the disaster, the saturated loess layer of liquefaction was mainly distributed about 15 m below the terrace surface, and the erosion volume in the source area is about 604,900 m^3^ (Fig. S10b).


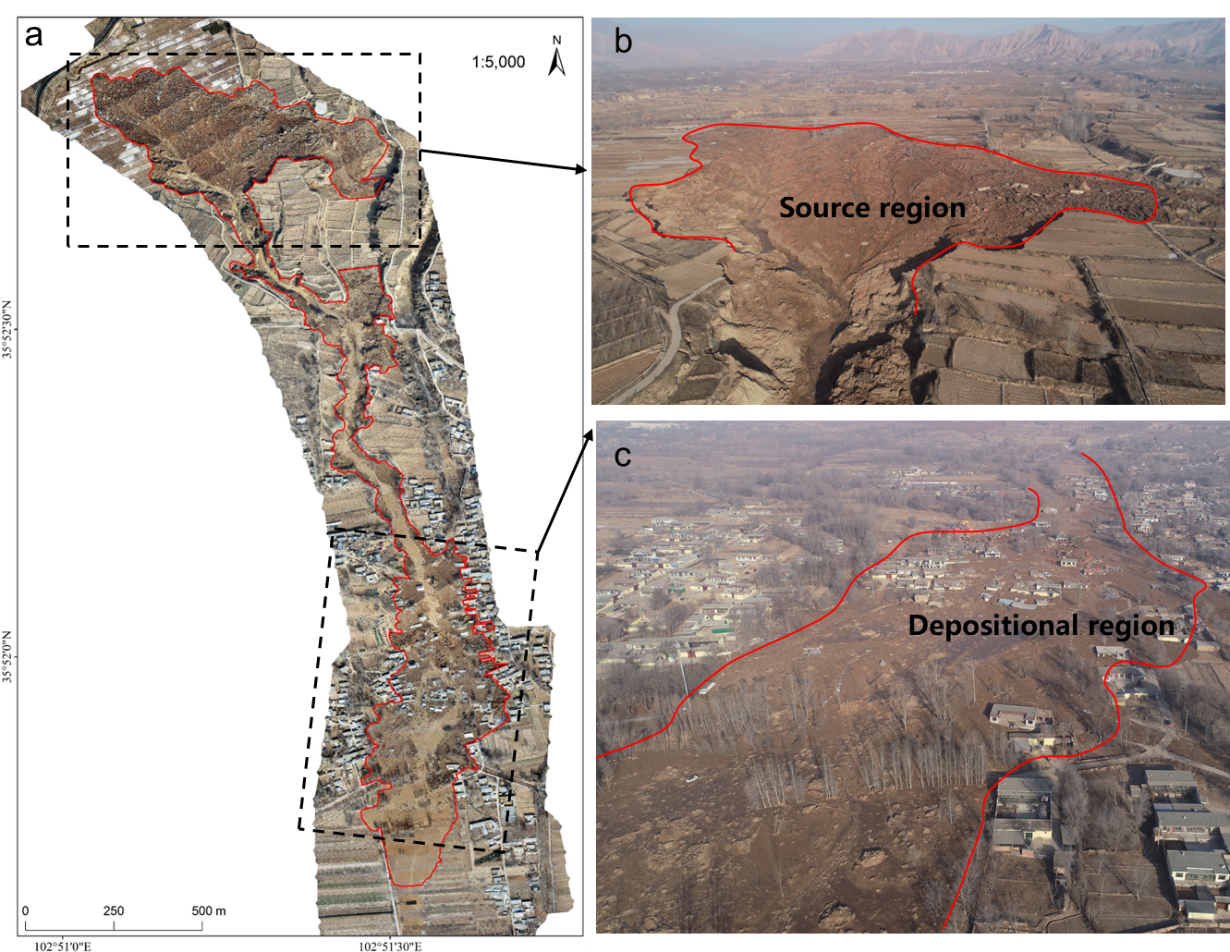


**Fig. S10 The Jintian and Caotan Village (JTCT) loess landslide-mudflow hazard induced by earthquake liquefaction (photograph by Meng Xingmin's team, December 27, 2023)**


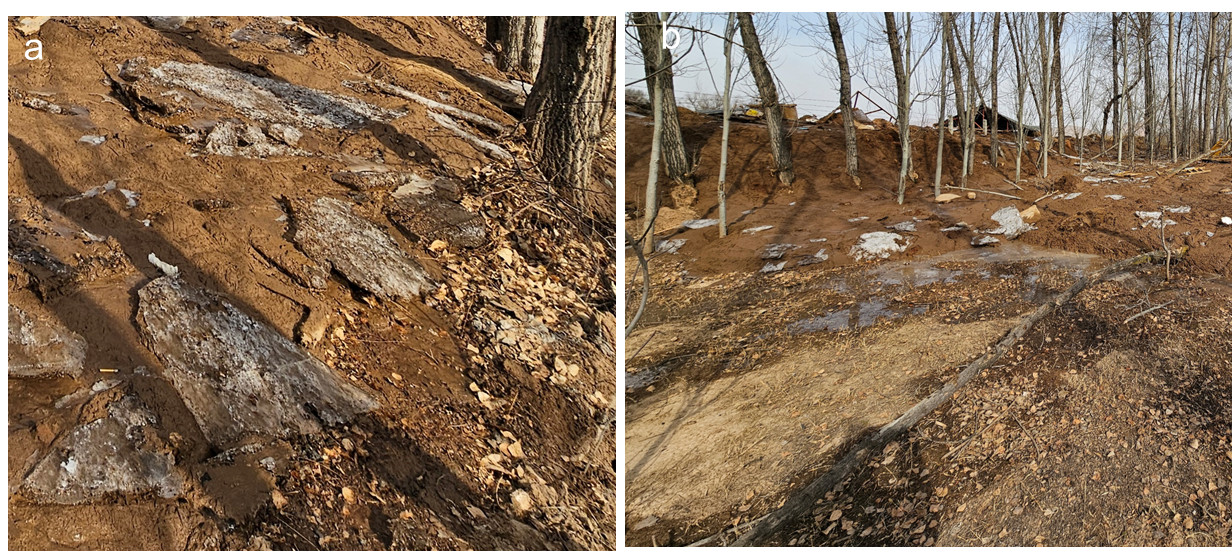


**Fig. S11 Ice blocks with a thickness of about 10–15 cm in the transport zone of the Ms 6.2 earthquake liquefied loess-landslide-mudflow hazard site in Jintian and Caotan Villages (JTCT), Zhongchuan Township, Minhe County, Qinghai Province (photograph by Meng Xingmin's team, December 27, 2023)**

**Supplement 6: Evidence on the similarity between deposit compositions of the Lajia Settlement and the Jintian and Caotan landslide and mudflow site in the same geological and geographic environment.**

On the December 18, 2023, a Ms 6.2 seismic event struck the Jishishan County, and the catastrophic seismic landslide and mudflow, named Jintian and Caotan landslide and mudflow, was induced in the Guanting Basin. The famous Lajia Settlement dated 4000a B.P. is only about 4.15 km to this site, and we argue that the cause of the destruction of the Lajia Settlement also followed a similar process. Professor Huang Chunchang's research team used the methods of sedimentology, chronology and paleoflood hydrology to examine that the red clay layer above the Qijia cultural layer at the Lajia site and found that the red clay deposit does not have the general characteristics of paleoflood deposits, but is a typical mudflow sediment^20,21,22,23^. Our understanding supports this view. Professor Zhou Qiang, one of the authors of this paper, has made a thorough and comprehensive analysis of the Quaternary geological geomorphology, sediology, chronology, palaeoflood hydrology, soil grain size, geochemical elements and UCC ratio curves of the Lajia site, and believes that the sediment that destroyed the Lajia settlement has the structure and composition of the weathered mudstone deposits of Paleogene that are very similar to those of Dahonghillside in the north of the Lajia Settlement, and are quite different from the palaeoflood sediments near the Lajia Settlement^24^. However, due to the limitations in understanding at that time, the cause of destruction of the Lajia Settlement was inferred to be a "flash flood mudslide" caused by an earthquake and heavy rain^24^. By a comprehensive comparison of the Holocene bottom sequence of Jintian and Caotan Villages and the Holocene bottom sequence of the Lajia Settlement site, We found that the two places have the same stratigraphic sequence (Fig. S12), indicating that the two places are not only have close spatial proximity to each other, but also have the same sedimentary process, the same geological development process, and both have the development of ancient mudflows.


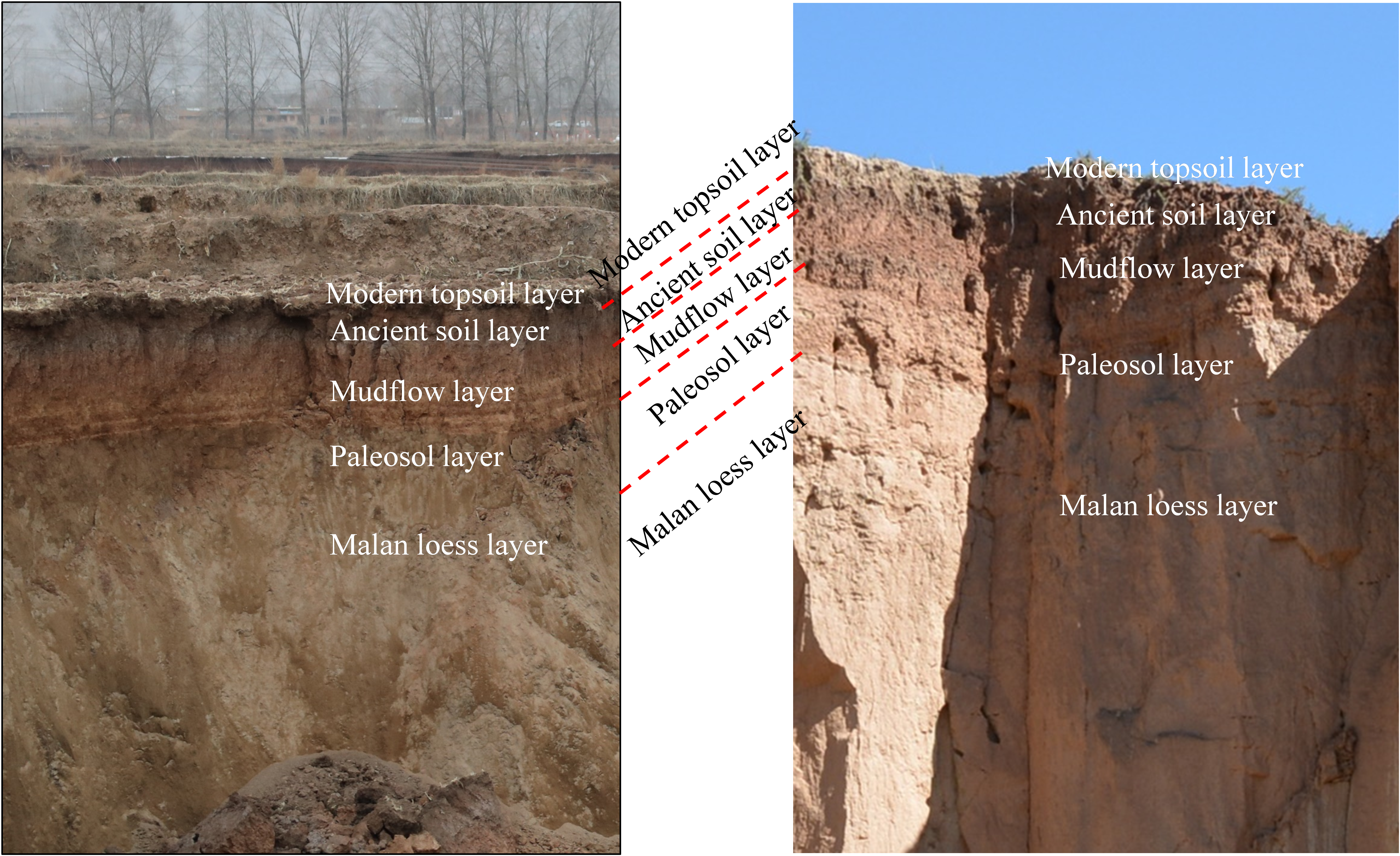


**Fig. S12** **Comparison between the stratigraphic profiles of Jintian and Caotan Villages (left) and the Lajia Settlement site (right) (photograph by Liu Fenggui's team)**

By comparing the macro features of the ancient flood sediments of the Yellow River and mudflow layers at the Lajia site and the Jintian and Caotan mudflow site (Fig. S13), we found that the paleoflood sediments in the Jishixia section of the Yellow River have a prominent rhythmic block structure, but the mudflow layers at the Lajia site and the Jintian and Caotan mudflow site have great similarities, both of which are characterized by an unoriented arrangement of particulate matter, showing an irregular wave-like flow structure, and very chaotic microscopic morphology, with a large number of voids and bubbles that show an irregular distribution. The results indicate that the mudflows changed the original particle distribution characteristics of the sediment in the source area during the flow process, and the mudflow head strongly disturbed, rolled, and flipped and mixed into a large amount of air. Therefore, based on the macroscopic features of sedimentary layers, we believe that the origin of the mudflow at the Lajia site is similar to that of the Jintian and Caotan mudflow, and it is very different from the flood sediment of the Yellow River.


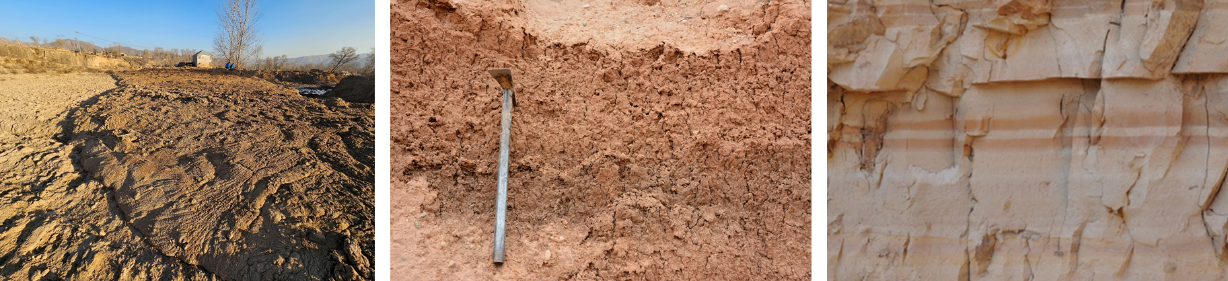

**Fig. S13 Comparison of the macro features of the Jintian and Caotan mudflow (left), Lajia mudflow layer (middle), and Yellow River paleoflood sediment layer (right) (photograph by Liu Fenggui's team)**

From the analysis of sediment particle size characteristics (Fig. S14), we found that the particle size distribution curves of the Jintian and Caotan mudflow (by Meng Xingmin's) and the mudflow sediment at the Lajia site^25,26^ have similar characteristics, both of which are unimodal. The Jintian and Caotan mudflow just occurred and was not affected by external forces, organisms and humans activities, and its peak particle size is slightly larger than that of the mudflow sediment at the Lajia Site. However, based on the distribution characteristics of both, we can infer the similarity between the origins of the mudflow at the Lajia Site and Jintian and Caotan mudflow. Accordingly, we made this supplementary discussion, and use Fig. S14 and the photos of sampling points as supplementary materials（Fig. S15）


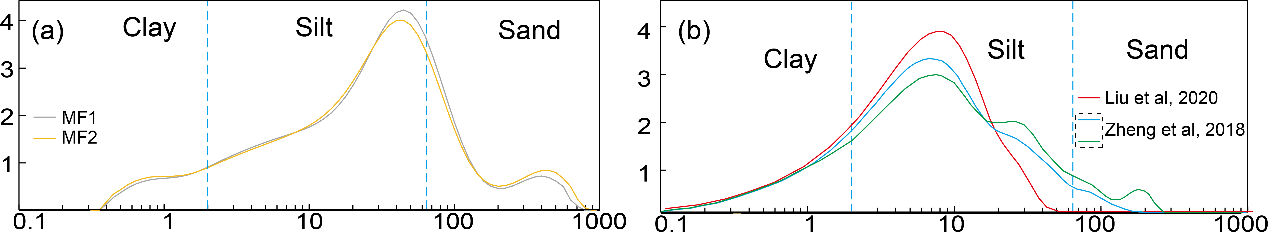


**Fig. S14 (a) Jintian and Caotan mudflow particle size distribution curve (by Meng Xingmin's team); (b) Mudflow particle size distribution curve of the Lajia site^25,26^. MF1 and MF2 are sampling sites.**


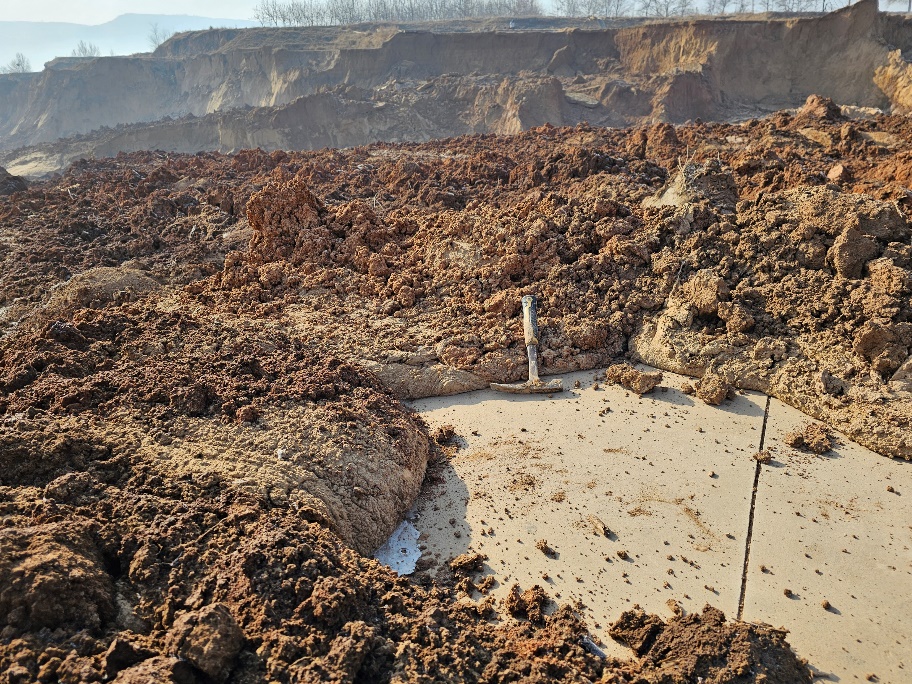

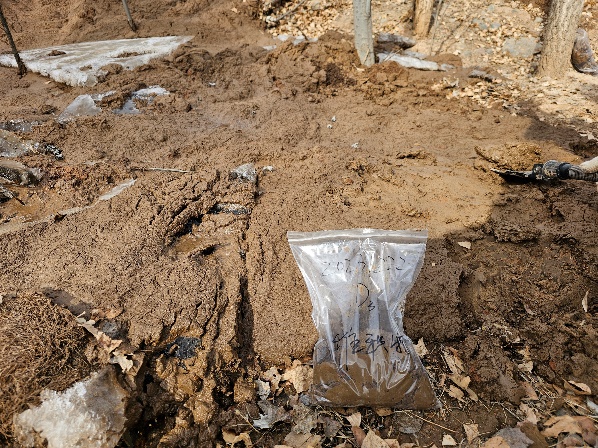


**Fig. S15 Photos of mudflow sampling sites (left: MF1, upstream; Right: MF2, downstream) (by Meng Xingmin's team)**

**Supplement 7: Photos of sampling points showing Jishishan earthquake ripple hazard**

Due to the large scale of the ripple hazard associated with the Jishishan earthquake, it is difficult to show the entire area involved in the disaster in a single picture. However, in the Jishishan earthquake, these hazards were most prominent in the mudflow source area of Jintian and Caotan Villages. We added on-site photos in the Supplement Materials document to support our view (Figs. S16-S19).


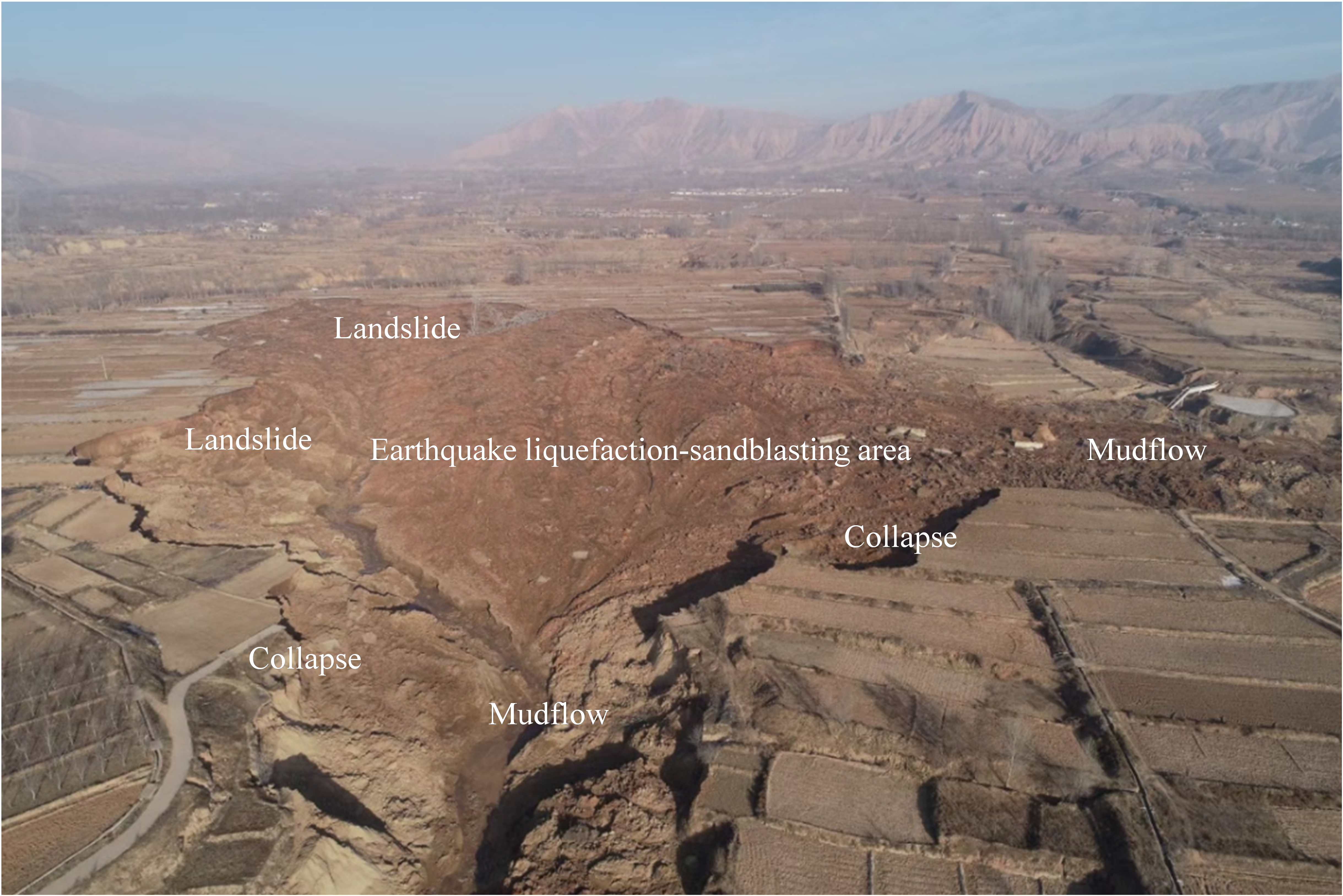


**Fig. S16 Local map of earthquake ripple hazard in the mudflow source area of Jintian andCaotan Villages (by Meng Xingmin's team)**


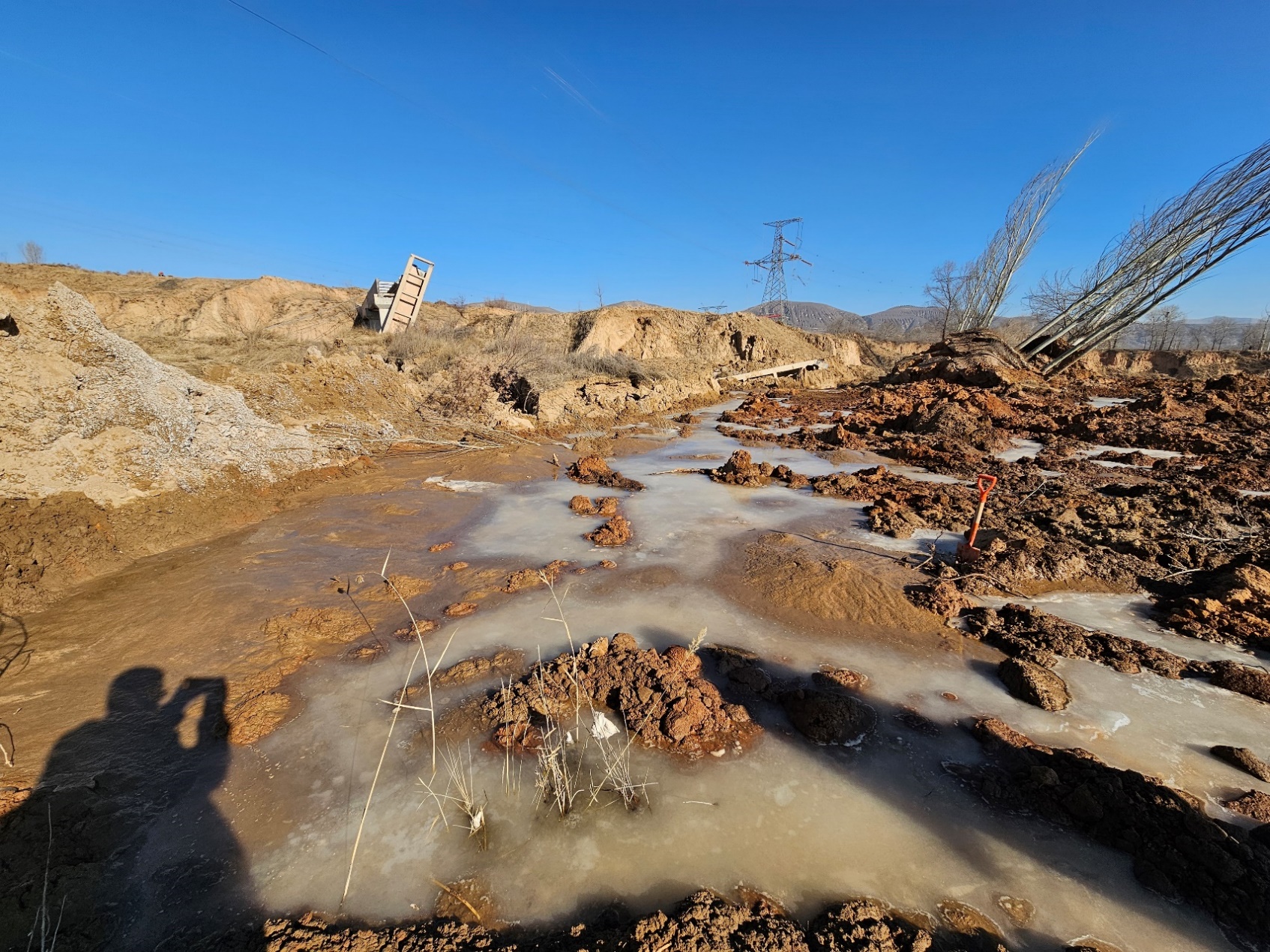


**Fig. S17 Field sandblasting in Jintian and Caotan Villages (by Meng Xingmin's team)**
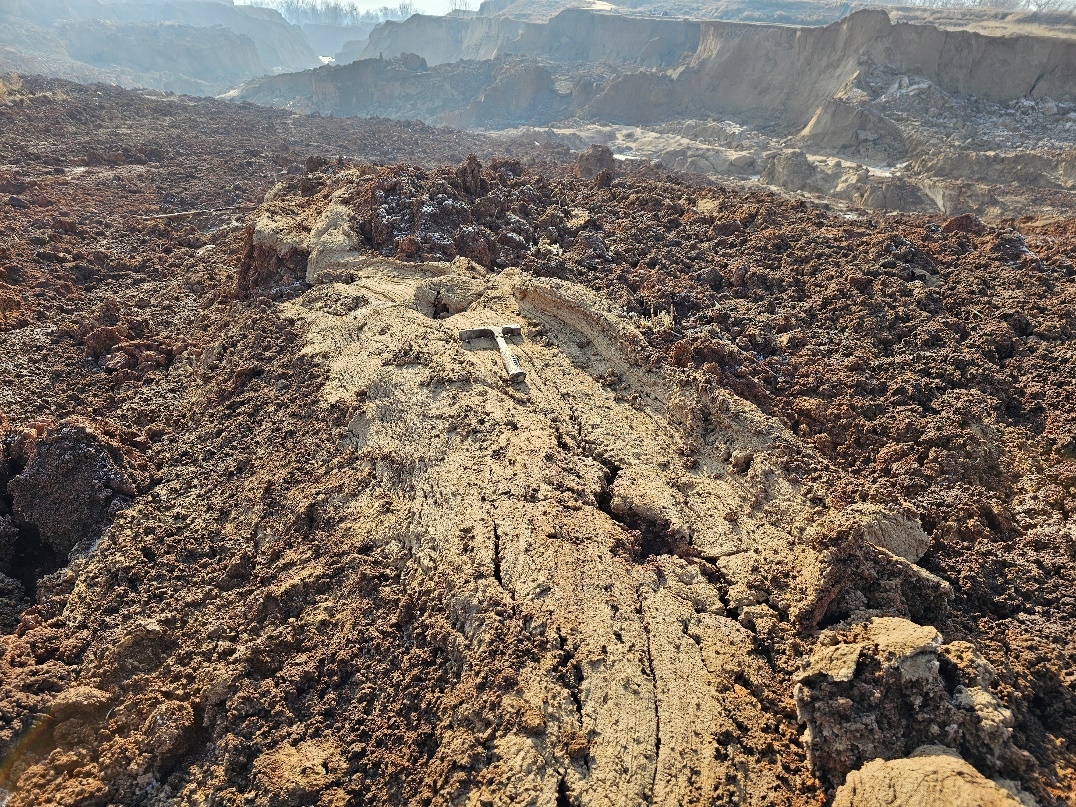


**Fig. S18 Soil liquefaction in Jintian and Caotan Villages (by Meng Xingmin's team)**
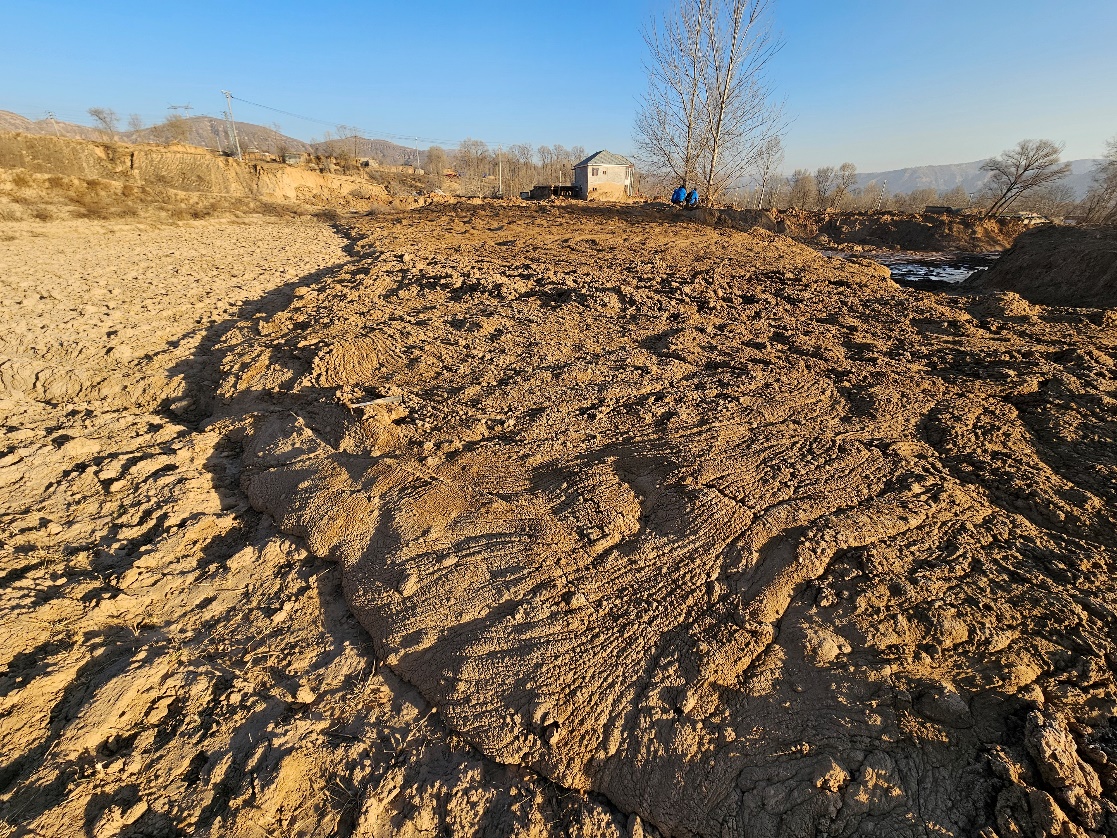


**Fig. S19 Flow pattern of the mudflow in Jintian and Caotan Villages (by Meng Xingmin's team)**

**Reference**

1. Shi, P. Disaster Risk science, pp.66-73 (BNUP and Springer, 2018).
2. Huang, C., Pang, J., Zhou, Y., et al. Palaeoenvironmental Implications of the Prehistorical Catastrophes in Relation to the Lajia Ruins within the Guanting Basin along the Upper Yellow River, China. The Holocene 23, 1584-1595 (2013).
3. Zhang, Y., Huang, C., Pang, J., et al. A luminescence dating study of the sediment stratigraphy of the Lajia Ruins in the upper Yellow River valley, China. Journal of Asian Earth Sciences 87, 157-164 (2014).
4. Zhang, Y., Huang, C., Pang, J., et al. OSL dating of the sediment stratigraphy of the Lajia Ruins in the Guanting Basin in the Upper Yellow River Basin. Acta Geographica Sinica 68, 626-639 (2013). (in Chinese)
5. Zhang, Y., Huang, C., Pang, J., et al. Micromorphology of ancient plow layer of paleosol in the Lajia Ruins in the Guanting Basin, Minhe County, Qinghai Province. Acta Pedologica Sinica 52, 1002-1013 (2015). (in Chinese)
6. Zhou, Q., Zhang, Y., Exploration and analysis of the reasons for pre-historical catastrophes in the Lajia Ruins, Qinghai Province, Acta Geographica Sinica 70, 1774-1787 (2015). (in Chinese)
7. Zheng, Z., Huang, C., Zhao, H., Guo, Y., Zhou, Y. Geochemical Characteristics of the Mid-Holocene Palaeosol and Mudflow Deposits in the Lajia Ruins of Qinghai Province. Mountain Research 36, 1-12 (2018). (in Chinese)
8. Liu, L. Pang, J., Huang, C., et al. Sediment Stratigraphy of the Paleo-pluvial Fans in the Guanting Basin along the Upper Yellow River. Acta Sedimentologica Sinica 38, 1239-1248 (2020). (in Chinese)
9. Li, J., Zhang, X., Yu,H., et al. Lajia Ruins in Qinghai Province Might Be Caused by Seismic-inducedLiquefaction Mudflows of Sands and Soils: Implication from the Jishishan Earthquake in Gansu Province on December 18, 2023. Geoscience 38, 258-269 (2024). (in Chinese)
10. Dong, G., Zhang, F., Liu, F., Zhang, D., et al. Multiple evidences indicate no relationship between prehistoridisasters in Lajia site and outburst flood in upper Yellow River valley, China. Science China Earth Sciences 61, 441-449 (2018). (in Chinese)
11. Li, Z., Li, Y., Tian, Q., et al. Study on the Relationship between Paleoseismic on Laji Mountain Fault and Catastrophic Event on Lajiashan Site. Journal of Seismological Research 37, 109-115 (2014). (in Chinese)
12. Xia, Z., Yang, X., Ye, M. Prehistoric disaster event at Lajia Site in Qinghai. Science bulletin 48, 1200-1204 (2003). (in Chinese)
13. Wu, Q., Zhao, Z., Liu, L., et al. Outburst flood at 1920 BCE supports historicity of China’s Great Flood and the Xia Dynasty. Science 353, 579-582 (2016).
